# Genome sequence of *Klebsiella pneumoniae* jumbo phage vB_KpnM_Magnus

**DOI:** 10.1128/mra.00977-26

**Published:** 2026-10-19

**Authors:** Ryan Walker-Gray, Danielle J. Ingle, Louise M. Judd, Eric Hanssen, Kathryn E. Holt, Zoe A. Dyson

**Affiliations:** 1 Department of Infection Biology, Faculty of Infectious and Tropical Disease, London School of Hygiene & Tropical Medicine 4906, London, United Kingdom; 2 Department of Microbiology and Immunology, Peter Doherty Institute for Infection & Immunity, University of Melbourne 2281, Melbourne, Victoria, Australia; 3 Centre for Pathogen Genomics, Department of Microbiology and Immunology, University of Melbourne 2281, Melbourne, Victoria, Australia; 4 Department of Biochemistry and Pharmacology, Bio21 Institute, The University of Melbourne 2281, Melbourne, Victoria, Australia; 5 Ian Holmes Imaging Centre, Bio21 Institute, The University of Melbourne 2281, Melbourne, Victoria, Australia; 6 Department of Infectious Diseases, School of Translational Medicine, Monash University 2541, Melbourne, Australia; 7 Wellcome Sanger Institute, Wellcome Genome Campus 47665, Hinxton, United Kingdom; Portland State University, Portland, Oregon, USA

**Keywords:** jumbo phage, bacteriophage, phage, *Klebsiella*, *Klebsiella pneumoniae*, myovirus, eowynvirus

## Abstract

We present the genome of phage vB_KpnM_Magnus, a jumbo phage infective for *Klebsiella pneumoniae* strain INF359 isolated from a wastewater sample from Melbourne, Australia. The genome sequence is 269,722 bp, with a G + C content of 45.64% and 377 genes.

## ANNOUNCEMENT

Bacteriophages with genomes >200 kbp are jumbo phages (1, 2). Jumbo phages infecting *Klebsiella pneumoniae*, a recognized World Health Organization priority pathogen, are understudied, as their isolation is technically challenging because their large size inhibits filtration methods used to remove host bacteria during culturing and lowers diffusion on agar, limiting visible plaque formation (3–5).

Phage vB_KpnM_Magnus was isolated via enrichment culture conducted at 37°C overnight using Luria-Bertani broth (Fisher Scientific; 25 mL), wastewater (5 mL; 0.22 μM filtered mixed liquor, Melton Recycled Water Plant, Australia, 16 August 2017), and exponential phase *K. pneumoniae* strain INF359 (200 μL; ST17, capsule type K25) (6–8). Using 0.4% Luria-Bertani agarose overlays (Fisher Scientific) (9), a lawn of INF359 was inoculated, exposed to a 50 μL drop of filtered (0.22 μM) enrichment culture, and incubated at 37°C overnight. Single plaques were excised and resuspended in 500 μL of Luria-Bertani broth, followed by purification through five rounds of dilution and reisolation (7). Phage vB_KpnM_Magnus produced clear plaques ~0.5 mm in diameter. Phage lysate (4 μL) was added to a glow discharge lacey carbon-coated copper grid, blotted, and plunge-frozen in liquid ethane, prior to visualization with a FEI Tecnai F30 electron microscope operating at 200 kV under low dose conditions (2,000 electron/nm^2^).

Five milliliters of phage lysate was subjected to RNAse and DNAse treatment, followed by polyethylene glycol 8000 precipitation, prior to gDNA extraction with a GenFind V3 DNA extraction kit (Beckman Coulter) (7, 10). Unsheared gDNA was prepared for sequencing with Nextera XT library and barcoding kits (Illumina) and sequenced via the MiSeq platform. Adapter trimming and demultiplexing were performed using BaseSpace v5.0. Raw 150 bp paired-end reads were assembled using Unicycler v.0.5.0 (11), yielding a consensus contig with >344-fold read depth. Genome annotation was performed using PHAROKKA v1.9.1 (12), followed by Phold v1.2.5 (13). Phage lifestyle was assessed using PhaTYP v2.1.13 (14), and taxonomic classification was performed using TaxMyPhage v3.3.6 (15). Genome structure was assessed by mapping raw reads to the assembled contig with Bowtie v2.2.4 (16), followed by manual inspection using IGV v2.17.4 (17). Default parameters were used for all bioinformatic analyses.

**Fig 1:**
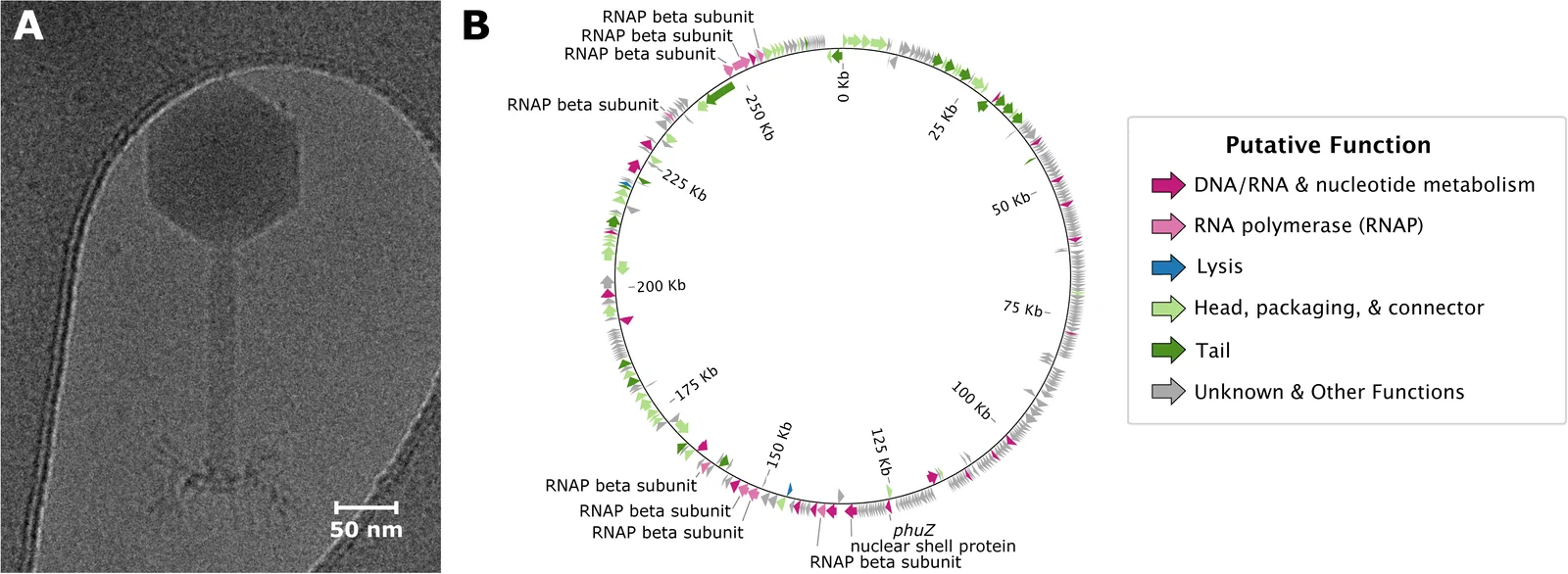
Morphology and genetic features of *Klebsiella* phage vB_KpnM_Magnus. (**A**) Cryo-transmission electron micrograph demonstrating myovirus morphology. (**B**) Genome map highlighting key genetic features. Arrows indicate coding sequences colored by putative function as per the inset legend. Key genetic features are labeled.

Phage vB_KpnM_Magnus possesses a myovirus morphology with complex tail fibers characteristic of some jumbo phages that infect a broad range of bacteria (Fig. 1A) (18). Its 269,722 bp genome is circularly permuted, with a G + C content of 45.64% and 377 putative genes. Phage vB_KpnM_Magnus appears to be an obligately lytic member of the genus *Eowynvirus*, sharing 78.2% average nucleotide identity with *Klebsiella* phage vB_KvM-Eowyn (accession LR881104), the only other member of this genus.

Jumbo phages have a large repertoire of genes that encode several characteristic traits also identified in phage vB_KpnM_Magnus (Fig. 1B). These include genes encoding a nuclear shell protein (CDS_237) and a PhuZ tubulin-like protein (CDS_219). These are involved in the formation and positioning of nucleus-like structures that shield phages from restriction-modification and CRISPR-Cas systems involved in host defense (19–24). Similarly, vB_KpnM_Magnus encodes two clusters of RNA polymerase (RNAP) β subunits (CDS_238, CDS_251, CDS_252, CDS_261, CDS_335, CDS_345, CDS_346, and CDS_351) reported to colocalize with the phage nucleus where they facilitate transcription, alleviating reliance on host machinery (25). Intriguingly, 69% of putative genes identified were of unknown function, and further studies are required to elucidate the potential role of jumbo phages in *K. pneumoniae* evolution and biology.

## Data Availability

The assembled and annotated sequence of phage vB_KpnM_Magnus has been deposited in GenBank under accession number PZ736737. Raw reads have been deposited in the Sequence Read Archive under accession number SRR39107557.
